# Dedicated Education Unit is a cost-effective clinical education model for undergraduate nursing programs

**DOI:** 10.31744/einstein_journal/2020GS5328

**Published:** 2020-06-17

**Authors:** Wendel Mombaque dos Santos, Rasika Jayasekara

**Affiliations:** 1 Escola de Enfermagem Universidade de São Paulo São PauloSP Brazil Escola de Enfermagem, Universidade de São Paulo, São Paulo, SP, Brazil.; 2 University of South Australia AdelaideSA Australia University of South Australia, Adelaide, SA, Australia.

**Keywords:** Costs and cost analysis, Economics, Nursing education research, Education

## Abstract

**Objective:**

To evaluate the cost-effectiveness of clinical education models for undergraduate nursing programs.

**Methods:**

A model-based cost-effectiveness analysis. Settings were universities with undergraduate nursing courses. Participants consisted of the decision tree that guided the structure of the model, filled in with effectiveness results from a hypothetical cohort of undergraduate nursing students. Interventions were Clinical Preceptor or Clinical Facilitator or Clinical Education Unit. Main outcome measure was effectiveness, defined as improvement of clinical education. The projected economic outcomes included incremental costs, incremental effectiveness, and incremental cost-effectiveness ratio. Monte Carlo probabilistic sensitivity analysis was employed to assess uncertainty in the model and robustness of our results.

**Results:**

The model based on Clinical Education Unit could be defined as the best, followed by Clinical Facilitator and Clinical Preceptor. The incremental cost of telephone-support intervention was US$ 59,604.40 higher than the second-best performing intervention (Clinical Facilitator), and US$ 32,661.86 higher than the last best performing intervention (Clinical Preceptor). In addition, Clinical Education Unit model showed 7% and 19% more effectiveness than Clinical Facilitator and Clinical Preceptor, respectively.

**Conclusion:**

Clinical Education Unit represents the best choice to promote better development of skills, knowledge and socialization in undergraduate nursing programs considering its effectiveness and costs.

## INTRODUCTION

Health care services and education organizations have currently sought alternatives to optimize learning of students.^(1)^ In the nursing context, the traditional model of clinical instruction predominates and, in many cases, has remained unchanged for decades.^(2-5)^ Although this model had been enough for decades, recent trends in education, health systems, and care of patients require that nursing education programs investigate innovative clinical teaching models to ensure optimal student preparation for practice.^(6-8)^

In this context, there is evidence that the clinical education model promotes a better development of skills, knowledge and socialization.^(9)^ Clinical education models were developed to improve clinical learning of future nurses, resulting in better quality of care provided to patients.^(1)^ The clinical education model is based on patient’s total care experiences, permeating a project of learning activities and adequate skills at undergraduate level.^(3,4,10-12)^

Although there is evidence evaluating various clinical education models for nursing undergraduate students, no attention has been given to the cost-effectiveness ratio of these models. Thus, there is a clear need for a complete cost-effectiveness assessment to examine the effectiveness of different models of clinical education, considering their costs and providing the best evidence available, so that managers of education organizations can choose the model that best fits in their financial scope.

## OBJECTIVE

To evaluate the cost-effectiveness of clinical education models for undergraduate nursing programs.

## METHODS

### Study design and patients

This study is a cost-effectiveness analysis comparing models to improve clinical education in undergraduate nursing programs, conducted at University of South Australia, Australia, in December 2018. It was carried out according to the recommendations of the Second Panel on Cost-Effectiveness in Health and Medicine.^(13)^ The analysis was also performed from the perspective of the Australian Education System (payer perspective). The result of this analysis was expressed as a ratio of incremental costs and incremental health intervention outcomes. The incremental cost-effectiveness ratios (ICER) were calculated in American dollars, in 2018.

### Interventions and model structure

The models of clinical education in undergraduate nursing programs define the model structure. They are Clinical Preceptor, Clinical Facilitator, and Clinical Education Unit (CEU).^(1)^

Clinical Preceptor involves assignment of students to practice, for a defined period, with experienced clinicians employed in the clinical facility. In the Clinical Facilitator model, healthcare workers (Clinical Facilitator) are employed by the education organizations to oversee aspects of the clinical placement for undergraduate nursing students across different clinical venues, including offering direct supervision and evaluation. Clinical Facilitators are experienced clinicians, mostly seconded from the hospital to the university. The CEU or Dedicated Education Unit (DEU) is a health care unit, developed by lecturers and clinicians, dedicated to the clinical education of nursing students. In Australia, a university collaborated with healthcare units to design a DEU that provides clinical placements of undergraduate nursing students during any year of the program.

### Model inputs

We derived model inputs from one systematic review^(1)^ that evaluated the effectiveness of clinical education models for undergraduate nursing programs and additional literature searches.

Costs were simulated based on the hour value of the professionals involved during the whole course of the nursing students. The values are measured by the student training cycle (considering the beginning and end of the undergraduate program). The cost was obtained from an estimate of the syllabus of the School of Nursing and Midwifery (University of South Australia).

Cost and effectiveness outcomes were discounted by 5%. Discounting was used in sensitivity analyses assessing differential effectiveness between strategies. All data of model inputs are present in table 1.

**Table 1:** Estimated parameter for economic cost-effectiveness analysis

Parameter	Distribution parameters (range)	Distribution	Sources
Baseline parameters			
Clinical Preceptor (improve education)	0.46 (0.44; 0.58)	Normal	Jayasekara et al.^(1)^
Odds ratio			
Clinical Preceptor	1.00	Normal	Jayasekara et al.^(1)^
Clinical Facilitator	2.77	Normal	Jayasekara et al.^(1)^
Clinical Education Unit	6.45	Normal	Jayasekara et al.^(1)^
Direct costs			
Clinical Preceptor	80,000 (-30%; +30%)	Triangular	Estimate based on University of South Australia
Clinical Facilitator	110,000 (-30%; +30%)	Triangular	Estimate based on University of South Australia
Clinical Education Unit	160,000 (-30%; +30%)	Triangular	Estimate based on University of South Australia

### Cost-effectiveness analysis

Effectiveness was defined as advance of clinical education by improving clinical decision-making and critical thinking skills.^(1)^ The three clinical education models for undergraduate nursing programs included in this analysis conferred statistically significantly improved clinical education, as compared to the Clinical Preceptor. The Clinical Preceptor was used for reference.

The projected economic outcomes included incremental costs, incremental effectiveness, and incremental cost-effectiveness ratio. We did not use a cost-effectiveness threshold. Results of cost-effectives analysis will be classified as possibly cost-effective (intervention more effective and less costly than the next least costly intervention), weakly dominated (intervention less effective, but has a smaller cost than the next highest ranked intervention), and dominated (intervention less effective and with a higher cost than the next least costly scenario).^(14)^

### Sensitivity analysis

Monte Carlo probabilistic sensitivity analysis was employed to assess uncertainty of the model and robustness of our results. We ran our model 100,000 times to estimate the mean costs and effectiveness, and used an informal method to produce equal distributions in a formal Bayesian analysis with uninformative priors.^(15)^

## RESULTS

**Figure 1:**
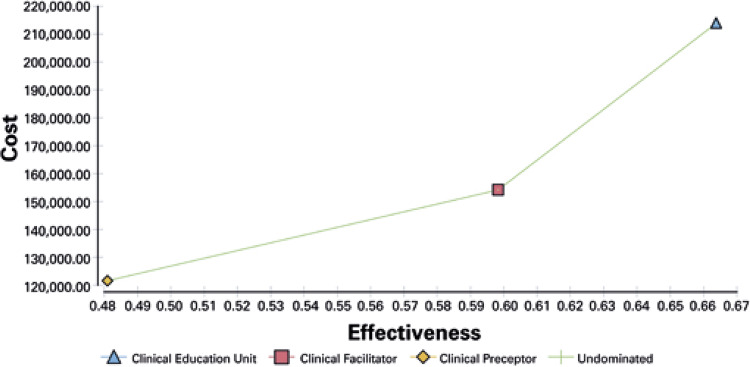
Cost-effectiveness analysis

Our base-case results are presented in figure 1. They show that across all interventions to improve clinical education in undergraduate nursing students, we have three possibly cost-effective interventions. The model based in CEU could be defined as the best, followed by Clinical Facilitator and Cinical Preceptor. Telephone-support intervention incremental cost was US$ 59,604.40 higher than the second-best performing intervention (Clinical Facilitator), and US$ 32,661.86 than the last best performing intervention (Clinical Preceptor). In addition, CEU model shows 7% and 19% more effectiveness than Clinical Facilitator and Clinical Preceptor, respectively.

The ICER of Clinical Facilitator compared to Clinical Preceptor was US$ 278,271.66, and the ICER of CEU comparing to Clinical Preceptor was US$ 909,825.50 per percent increase in clinical education (Table 2).

**Table 2:** Estimated cost, effectiveness, incremental cost-effectiveness, net monetary benefit and cost-effectiveness analysis of interventions to improve clinical education

Strategy	Cost (US$)	Effectiveness	ICER	Interpretation
Clinical Preceptor	121,528.00	0.48	-	Cost-effective
Clinical Facilitator	154,189.90	0.59	278,271.66	Cost-effective
Clinical Education Unit	213,794.30	0.66	909,825.50	Cost-effective

ICER: incremental cost-effectiveness ratio.

**Figure 2:**
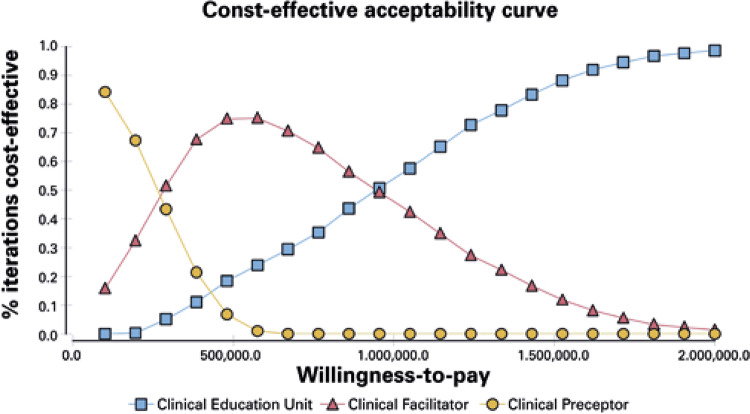
Probabilistic sensitivity analysis

The probabilistic sensitivity analysis verified that our base-case cost-effectiveness analysis was robust. The probabilistic sensitivity analysis showed, in the most hypothetical scenarios, the intervention based in CEU is the best choice, considering a willingness-to-pay of 1,000,000 (Figure 2).

## DISCUSSION

This model suggested that CEU represents an additional effectiveness and cost to education models for undergraduate nursing programs, when compared to Clinical Facilitator or Clinical Preceptor.^(6)^

The CEU promotes learning and allows time and space for reflection, besides developing a professional group identity, and learning to recognize and implement the responsibilities related to the nurse professional role.^(7)^ An educational model focused on education organizations, as herein considered, is likely to reduce duplication of costs and results in savings, when considering the different undergraduate courses using CEU. However, the model assumed that the costs were unique to the undergraduate nursing course, regardless of the number of students. In the future, models involving the maintenance of different undergraduate courses can be developed.

The cost to implement and maintain CEU was significantly higher than the tradition model (Clinical Preceptor).^(4,12,16)^ However, no study was able to determine the effectiveness and long-term cost when these undergraduate students would be working as nurses. We know that as an educational administrator, it is very attractive to have a model that allows training more students with similar results and at a lower financial cost. However, the current focus should be on the quality that these professionals can provide while they are working. The costs of complications arising from inadequate health care can be much higher than the investment in setting up and maintaining a CEU.

Educational factors have limited the number of undergraduate nursing students in advanced practice and consequent delay in training human resources.^(5,10,11)^ Aiming to assist in the shortage of nurses, health services are challenged to release part of the nursing team to become supervisors in clinical supervision models, and require a significant investment in nurses’ education to achieve adequate teaching effectiveness.^(5,10,11)^ In this way, CEU provides a simulation, and the academic-practice partnership model can offer innovative approaches to clinical training, aiming to produce graduates who can provide safe and quality care services within the complex environment based on system practice of health.^(1,5,10,11)^

This study has some limitations. However, we should emphasize that the long-term advantages of CEU can be even greater, since professionals that are more qualified improve patients’ clinical outcomes, reduce costs and have better productivity.

## CONCLUSION

This analysis demonstrated that Clinical Preceptor, Clinical Facilitator and Clinical Education Unit are cost-effective models for clinical education of undergraduate nursing students. However, the probabilistic sensitivity analysis showed that, in the most hypothetical scenarios, the intervention based on Clinical Education Unit is the best choice. This suggests that Clinical Education Unit represents the best choice to promote better development of skills, knowledge and socialization for undergraduate nursing programs, considering effectiveness and costs. Nursing programs should encourage the implementation of Clinical Education Unit, so that the training of professionals will be more appropriate to the real needs of patients.
