# Supplementary material for: Dedicated Education Unit is a cost-effective clinical education model for undergraduate nursing programs
Source: Einstein (Sao Paulo). 2020 Jun 17;18:eGS5328. doi: 10.31744/einstein_journal/2020GS5328 (PMC7299527; doi:10.31744/einstein_journal/2020GS5328)
Supplement: Translation

# Translation: A Unidade de Educação Dedicada é um modelo de educação clínica custo-efetivo para programas de graduação em enfermagem

**Authors:** Wendel Mombaque dos Santos, Rasika Jayasekara

**Affiliations:** 1 Brasil Escola de Enfermagem, Universidade de São Paulo, São Paulo, SP, Brasil.; 2 Australia University of South Australia, Adelaide, SA, Australia.

**Keywords:** Custos e análise de custo, Economia, Pesquisa em educação de enfermagem, Educação

## Abstract

**Objetivo:**

Avaliar a relação de custo-efetividade dos modelos de educação clínica para cursos de graduação em enfermagem.

**Métodos:**

Análise de custo-efetividade baseada em modelo. Os ambientes foram universidades com cursos de graduação em enfermagem. Os participantes consistiram na árvore de decisão, que norteou a estrutura do modelo, preenchida com resultados de efetividade de uma coorte hipotética de estudantes de graduação em enfermagem. As intervenções foram o Preceptor Clínico ou o Facilitador Clínico ou a Unidade de Educação Clínica. A principal medida de resultado foi a efetividade, definida como a melhoria da educação clínica. Os resultados econômicos projetados incluíram custos incrementais, efetividade incremental e custo incremental por efetividade. A análise probabilística de sensibilidade de Monte Carlo foi utilizada para avaliar a incerteza no modelo e a robustez de nossos resultados.

**Resultados:**

O modelo baseado na Unidade de Educação Clínica foi o melhor, seguido pelo Facilitador Clínico e pelo Preceptor Clínico. O custo incremental da intervenção com suporte por telefone foi US$ 59,604.40 a mais do que o da segunda intervenção de melhor desempenho (Facilitador Clínico) e US$ 32,661.86 a mais do que a última intervenção de melhor desempenho (clínico preceptor). Além disso, o modelo da Unidade de Educação Clínica mostrou 7% e 19% mais efetividade do que Facilitador Clínico e Preceptor Clínico, respectivamente.

**Conclusão:**

A Unidade de Educação Clínica representa a melhor escolha para promover um desenvolvimento de habilidades otimizado, conhecimentos e socialização nos cursos de graduação em enfermagem, considerando efetividade e custos.

## INTRODUÇÃO

Atualmente, os serviços de saúde e as instituições de ensino têm buscado alternativas para otimizar a aprendizagem dos alunos.^(1)^ No contexto da enfermagem, há um predomínio do modelo tradicional de instrução clínica que, em muitos casos, permanece inalterado há décadas.^(2-5)^ Embora esse modelo tenha sido suficiente por muito tempo, tendências mais recentes no ensino, nos sistemas de saúde e no atendimento aos pacientes exigem que os programas de formação de enfermeiros estudem modelos inovadores de ensino clínico, os quais devem garantir o melhor preparo possível para a prática profissional.^(6-8)^

Neste contexto, há evidências de que o modelo de educação clínica promove um melhor desenvolvimento de habilidades, conhecimentos e socialização.^(9)^ Os modelos de educação clínica foram desenvolvidos para melhorar a formação clínica dos futuros enfermeiros, levando a uma melhor qualidade de atendimento aos pacientes.^(1)^ O modelo de educação clínica baseia-se em experiências de assistência total ao paciente, permeando um projeto com atividades de ensino e habilidades apropriadas para o nível de graduação.^(3,4,10-12)^

Embora existam avaliações dos vários modelos de educação clínica de estudantes de enfermagem, não há análises específicas da relação de custo-efetividade desses modelos. Assim, existe claramente a necessidade de uma avaliação completa de custo-efetividade, examinando a efetividade dos diferentes modelos de educação clínica, considerando os respectivos custos e fornecendo as melhores evidências disponíveis, para que os gestores das instituições de ensino possam escolher o modelo que mais se encaixa em sua realidade financeira.

## OBJETIVO

Avaliar a custo-efetividade dos modelos de educação clínica para cursos de graduação em enfermagem.

## MÉTODOS

### Desenho do estudo e pacientes

Este estudo é uma análise de custo-efetividade que compara modelos para melhoria da educação clínica nos cursos de graduação em enfermagem, realizado na *University of South Australia*, Austrália, em dezembro de 2018. O estudo foi conduzido conforme as recomendações do *Second Panel on Cost-Effectiveness in Health and Medicine*.^(13)^ A análise também foi realizada na perspectiva do sistema australiano de educação (perspectiva do financiador). O resultado desta análise foi expresso como uma razão dos custos incrementais e dos resultados incrementais de saúde da intervenção. As razões de custo-efetividade incremental (RCEI) foram calculadas em dólares americanos, em 2018.

### Intervenções e estrutura do modelo

Os modelos de educação clínica dos cursos de graduação em enfermagem definem a estrutura do modelo. Eles são Preceptor Clínico, Facilitador Clínico e Unidade de Educação Clínica (UEC).^(1)^

O Preceptor Clínico envolve a nomeação de alunos para atuar, durante um período determinado, com clínicos experientes que trabalhem na instituição. No modelo de Facilitador Clínico, os profissionais de saúde (facilitadores clínicos) são funcionários das organizações de ensino, que supervisionam aspectos da colocação de estudantes de enfermagem em diferentes instituições clínicas, oferecendo, inclusive, supervisão e avaliação diretas. Os facilitadores clínicos são clínicos experientes, geralmente recomendados pelo hospital para a universidade. A UEC, ou Unidade de Educação Dedicada (UED), é uma unidade de saúde desenvolvida por acadêmicos e clínicos, dedicada à educação clínica dos estudantes de enfermagem. Na Austrália, uma universidade trabalhou junto das unidades de saúde para desenvolver uma UED encarregada pela colocação clínica de estudantes, em qualquer ano do curso de enfermagem.

### Dados de base para o modelo

Derivamos os dados de base do modelo de uma revisão sistemática,^(1)^ que avaliou a efetividade de modelos de educação clínica para cursos de graduação em enfermagem, além de buscas adicionais na literatura.

Os custos foram simulados com base no valor-hora dos profissionais envolvidos durante todo o curso dos estudantes de enfermagem. Os valores são medidos por ciclo de capacitação dos estudantes (considerando-se o início e o fim do curso de graduação). O custo foi obtido a partir de uma estimativa do programa curricular da *School of Nursing and Midwifery* (*University of South Australia*).

Descontamos 5% dos resultados de custo e efetividade. O desconto foi usado nas análises de sensibilidade que avaliaram a efetividade diferencial entre as estratégias. Todos os dados de base dos modelos são apresentados na tabela 1.

**Tabela 1:** Parâmetro estimado para a análise econômica de custo-efetividade

Parâmetro	Parâmetros de distribuição (variação)	Distribuição	Fontes
Parâmetros iniciais			
Preceptor Clínico (melhoria da educação)	0,46 (0,44; 0,58)	Normal	Jayasekara et al.^(1)^
Razão de chances			
Preceptor Clínico	1,00	Normal	Jayasekara et al.^(1)^
Facilitador Clínico	2,77	Normal	Jayasekara et al.^(1)^
Unidade de Educação Clínica	6,45	Normal	Jayasekara et al.^(1)^
Custos diretos			
Preceptor Clínico	80,000 (-30%; +30%)	Triangular	Estimativa com base na *University of South Australia*
Facilitador Clínico	110,000 (-30%; +30%)	Triangular	Estimativa com base na *University of South Australia*
Unidade de Educação Clínica	160,000 (-30%; +30%)	Triangular	Estimativa com base na *University of South Australia*

### Análise de custo-efetividade

Efetividade foi definida como aprimoramento da educação clínica, com melhores habilidades de tomada de decisões e pensamento crítico.^(1)^ Os três modelos de educação clínica para cursos de graduação em enfermagem incluídos na análise geraram melhoria na educação clínica com significância estatística, em comparação ao Preceptor Clínico. O Preceptor Clínico foi usado como referência.

Os desfechos econômicos projetados incluíram custos incrementais, efetividade incremental e custo incremental por efetividade. Não usamos limiar de custo-efetividade. Os resultados da análise de custo-efetividade serão classificados como possivelmente custo-efetivo (intervenção mais efetiva e com custo menor do que a próxima intervenção com menor custo), fracamente dominado (intervenção menos efetiva, mas com custo mais baixo que a próxima no *ranking*) e dominado (intervenção menos efetiva e com custo mais alto que o próximo cenário com custo mais baixo).^(14)^

### Análise de sensibilidade

A análise probabilística de sensibilidade de Monte Carlo foi usada para avaliar a incerteza do modelo e a robustez dos nossos resultados. Rodamos nosso modelo 100 mil vezes para estimar os custos e a efetividade média, e usamos um método informal para produzir distribuições iguais em uma análise Bayesiana formal com priores não informativos.^(15)^

## RESULTADOS

Os resultados de nossos casos de base são apresentados na figura 1. Eles mostram que, dentre as intervenções para aprimorar a educação clínica de alunos de graduação em enfermagem, temos três intervenções possivelmente custo-efetivas. O modelo baseado em Unidade de Educação Clínica foi considerado o melhor, seguido por Facilitador Clínico e Preceptor Clínico. O custo incremental da intervenção com suporte telefônico foi US$ 59,604.40 a mais do que o da segunda melhor opção (Facilitador Clínico) e US$ 32,661.86 a mais que o da terceira melhor opção (Preceptor Clínico). Além disso, o modelo de UEC é 7% e 19% mais efetivo que o Facilitador Clínico e o Preceptor Clínico, respectivamente.

**Figura 1:** Análise de custo-efetividade

A RCEI do Facilitador Clínico, comparada à do Preceptor Clínico, foi de US$ 278,271.66, e a RCEI da UEC comparada à do Facilitador Clínico foi de US$ 909,825.50 por aumento percentual na melhoria da educação clínica (Tabela 2).

**Tabela 2:** Custo estimado, efetividade, custo-efetividade incremental, benefício monetário líquido e análise de custo-efetividade de intervenções para melhoria da educação clínica

Estratégia	Custo (US$)	Efetividade	RCEI	Interpretação
Preceptor Clínico	121,528.00	0,48	-	Custo-efetivo
Facilitador Clínico	154,189.90	0,59	278,271.66	Custo-efetivo
Unidade de Educação Clínica	213,794.30	0,66	909,825.50	Custo-efetivo

RCEI: razão de custo-efetividade incremental.

A análise probabilística de sensibilidade verificou que nossa análise de custo-efetividade do caso base foi robusta. A análise probabilística de sensibilidade mostrou que, nos cenários mais hipotéticos, a intervenção baseada em UEC é a melhor opção, considerando disposição a pagar de 1,000,000 (Figura 2).

**Figura 2:** Análise probabilística de sensibilidade

## DISCUSSÃO

Este modelo sugere que a UEC traz um adicional de efetividade e custo para os modelos educacionais dos cursos de graduação em enfermagem, em comparação ao Facilitador Clínico ou ao Preceptor Clínico.^(6)^

A UEC promove aprendizagem e proporciona tempo e espaço para reflexão, além de ajudar no desenvolvimento de uma identidade de grupo e no reconhecimento e na implementação de responsabilidades relativas ao papel profissional do enfermeiro.^(7)^ Um modelo de ensino focado em instituições educacionais, conforme utilizado aqui, provavelmente reduz os custos duplicados e resulta em economia, quando se consideram os diferentes cursos de graduação que usam UEC. No entanto, o modelo pressupõe que os custos são inerentes ao curso de graduação em enfermagem, independentemente do número de alunos. No futuro, podem ser desenvolvidos modelos que envolvam a manutenção de diferentes cursos de graduação.

O custo de implementação e manutenção da UEC foi consideravelmente maior que o do modelo tradicional (Preceptor Clínico).^(4,12,16)^ Porém, nenhum estudo conseguiu determinar a efetividade e o custo de longo prazo, quando esses alunos de graduação já estivessem atuando como enfermeiros. Sabemos que, para os administradores de instituições de ensino, é muito interessante ter um modelo que permita formar mais alunos com resultados semelhantes e a um custo financeiro mais baixo. Porém, o foco atualmente deve ser na qualidade do trabalho desses profissionais quando estiverem atuando. Os custos das complicações decorrentes de serviços de saúde inadequados podem ser muito mais altos do que o investimento para a implementação e manutenção de uma UEC.

Fatores educacionais têm limitado o número de alunos de graduação em enfermagem com acesso à prática avançada e, consequentemente, gerado atrasos na capacitação de recursos humanos.^(5,10,11)^ Para auxiliar nessa escassez de enfermeiros, os serviços de saúde têm o desafio de liberar uma parte da equipe de enfermeiros para atuarem como supervisores em modelos de supervisão clínica, sendo necessário um investimento considerável na formação de enfermeiros, para atingir um nível de efetividade adequado.^(5,10,11)^ Assim, a UEC permite a simulação, e o modelo de parceria de prática acadêmica oferece abordagens inovadoras para a capacitação clínica, visando produzir graduandos que possam prestar serviços seguros e de qualidade, dentro do ambiente complexo baseado na prática sistêmica da saúde.^(1,5,10,11)^

Este estudo tem algumas limitações. No entanto, devemos destacar que as vantagens da UEC no longo prazo podem ser ainda maiores, já que profissionais mais bem qualificados levam a melhores desfechos para os pacientes, reduzem custos e apresentam maior produtividade.

## CONCLUSÃO

Esta análise demonstrou que Preceptor Clínico, Facilitador Clínico e Unidade de Educação Clínica são modelos custo-efetivos de educação clínica para alunos de graduação em enfermagem. No entanto, a análise de sensibilidade probabilística mostra que, nos cenários mais hipotéticos, a intervenção com Unidade de Educação Clínica é a melhor opção. A Unidade de Educação Clínica representa a melhor opção para um melhor desenvolvimento de habilidades, conhecimentos e socialização nos cursos de graduação em enfermagem, considerando-se efetividade e custo. Os cursos de enfermagem devem promover a implementação da Unidade de Educação Clínica, para que a capacitação dos profissionais seja mais adequada às necessidades reais dos pacientes.
